# Evolution of the Secondary Metabolites in Invasive Plant Species *Chromolaena odorata* for the Defense and Allelopathic Functions

**DOI:** 10.3390/plants12030521

**Published:** 2023-01-23

**Authors:** Hisashi Kato-Noguchi, Midori Kato

**Affiliations:** Department of Applied Biological Science, Faculty of Agriculture, Kagawa University, Miki, Kagawa 761-0795, Japan

**Keywords:** allelochemical, herbivore, invasive species, monospecific stand, natural enemy, nematode, pathogen

## Abstract

*Chromolaena odorata* (L.) R.M. King & H. Robinson is native to tropical America, and has naturalized in many other countries in tropical Asia, Austria, and West Africa. The species often forms dense thickets and reduces the native species diversity and population in the invasive ranges. The species is also considered as a noxious weed in agriculture fields, and listed in the 100 of the world’s worst invasive alien species. The characteristics of its life-history such as the seed production rate, growth pattern, and adaptative ability to the environmental conditions may contribute to the invasiveness of the species. Possible evidence of the defense capacity against the natural enemy, and the allelopathic potential against the competitive plant species for *C. odorata* has been accumulated in the literature over three decades. The extracts, residues, and/or rhizosphere soil of *C. odorata* increased the mortality of various insects and parasitic nematodes, and decreased their population. The extracts, residues, and/or rhizosphere soil of *C. odorata* also inhibited the germination and growth of several plant species including the indigenous plant species in the invasive ranges of *C. odorata.* Toxic substances, pyrrolizidine alkaloids were found in the leaves and flowers of *C. odorata.* These pyrrolizidine alkaloids may work as the defense agents against the natural enemies. Several potential allelochemicals such as flavonoids, phenolic acids, and terpenoids were also found in the plant extracts of *C. odorata*. Some of these compounds may work as allelopathic agents of *C. odorata* and inhibit the germination and growth of the competitive plant species. These characteristics of *C. odorata* for the defense function against their natural enemies such as insects and parasitic nematodes, and allelopathic potential against the competitive native plant species may contribute to the invasiveness and naturalization of *C. odorata* in the new habitats as invasive plant species. However, it is necessary to determine the concentration of these allelochemicals in the neighboring environment of *C. odorata* such as the rhizosphere soil since allelochemicals are able to work only when they are released into the neighboring environment. It is the first review article focusing on the defense function and allelopathy of *C. odorata*.

## 1. Introduction

**Figure 1:**
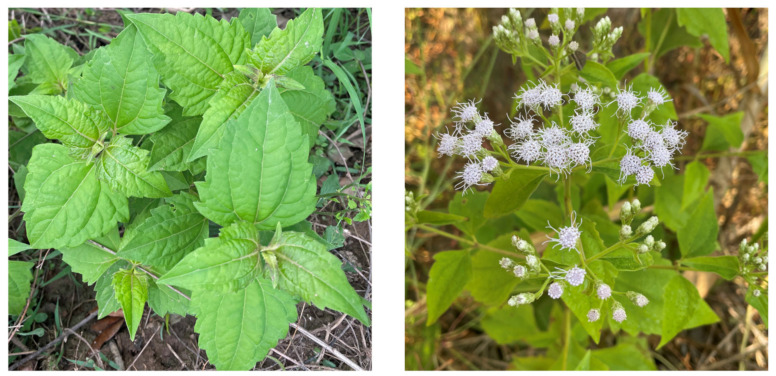
*C. odorata;* photos were kindly provided by Dr. Poonpaiboonpipat, T.

*Chromolaena odorata* (L.) R.M. King & H. Robinson (synonym, *Eupatorium odoratum* L.), belonging to Asteraceae, is a perennial shrub, and grows to 2–3 m in height, but it can scramble up other plants and reach 5–10 m in length. Its stems are cylindrical and pithy, become woody, and often branch in pairs from the axillary buds. The opposite leaves are ovate-triangular, 6–12 cm long and 3–7 cm wide with a 1–3 cm petiole, and smell a strong odor when they are crushed [1] (Figure 1). It has an abundantly branched lateral fine root system supported by corms which are underground swollen stems and storage nutrients [1,2,3]. Capitula are generated in panicles at the end of the twigs, and a single capitulum contains 15–35 florets. The corollas of the florets vary in color ranging from white to pale-lilac. The species grows on all types of well-drained soil, and where the temperature ranges 20–37 °C, and the minimum annual rainfall is 1500 mm [4,5]. However, it grows best in sunny and open areas such as roadsides, riverbanks, and vacant land [6,7,8].

The native range of *C. odorata* is from southern Florida and Texas (30° N) to north-western Argentina (30° S) including the Caribbean islands [9,10,11,12,13]. The species is thought to be introduced into Asia as an ornamental plant in 1840s through the Botanical garden in Kolkata, India. The first record of naturalization of *C. odorata* was in the 1870s in Indo-Gangetic Plain [1]. The species has also been introduced, spread rapidly, and naturalized in many other countries in eastern and southern Asia, Austria, and southern and western Africa [1,11,12].

*C. odorata* infests in a wide range of natural vegetation such as grassland, savanna, bush, forest margins and gaps, dry deciduous and degraded forests. The species scrambles up to the canopy of trees, and spreads over the trees, eventually reducing the vigor of the trees [14,15,16]. After infestation, it grows rapidly and often forms a dense thicket, extending its multiple branches and twisting them around the existing vegetation. The thickets of the species prevent the movement of the wildlife and livestock, which affects their breeding and population [1,17,18]. It was reported that the species reduced the biodiversity and population in its introduced vegetation [19,20]. Average number of the plant species in the infested areas of *C. odorata* was reduced by 31% [21]. *C. odorata* also infests as a weed in agricultural lands such as oil palm, rubber, cacao, coffee, coconut and banana plantations, pastures, crop fields, and abandoned agriculture fields. The species is considered as a noxious weed in agricultural fields and commercial plantations [22,23], and listed in the 100 of the world’s worst invasive alien species [11,12]. In its native ranges, *C. odorata* is only weedy on some occasions such as after the fire, hurricane, human activity, and other destruction events. It is then forced out by the successional vegetation and disappears [24,25]. However, the species forms dense monospecific stands and shrives over 15 years in the introduced ranges [1,26].

It was estimated that approximately 10% of the introduced plant species could be established in the introduced ranges, and 10% of the established plants become invasive [27], which indicates only 1% of the introduced plants could be invasive. The characteristics of life-history, such as the high reproduction rate, phenotypic plasticity, and competitive ability of the plants are important for the naturalization of invasive plants into the introduced ranges [28,29,30,31].

*C. odorata* is also a prolific seed producer. It was reported to produce 2000 (1 year old *C. odorata*)–260,000 (10 year old) seeds per m^2^ with 20–46% of seeds being viable. The germination rate of the seeds collected from the seed banks was 5–20% [26,32,33]. The foliage of the species, which contains essential oil, is flammable and increases the wild fire occurrence and its maximum temperature [34,35]. The intense fire increases the mortality of indigenous herbaceous plant species as well as the juvenile forest woody plants. The development of the stump sprouts of *C. odorata* from its underground stems, corms, arises quickly after the fire, and the regeneration of the species occurs [2,36,37]. Therefore, the wild fire may convert a native ecosystem of the woody plants and herbaceous into an *C. odorata* dominated ecosystem.

Genetic diversity of *C. odorata* in the introduced ranges was lower than that in the native ranges [38,39,40,41]. The morphological variability of *C. odorata* such as flower color, leaf shape, and plant shape is high in the native range, while there are only two main biotypes, Asia/West Africa biotype and South Africa biotype, in the invasive ranges [1]. Asia/West Africa biotype is thought to be originating from Trinidad and Tobago [40,42], while South Africa biotype is thought to be originating from Jamaica and/or Cuba [42,43]. However, the adaptative potential of the species was higher in the invasive range than that of the native ranges in the response to the environmental factors such as light conditions, mean annual temperature and precipitation [39,44,45]. Drought tolerance of the species in the invasive ranges was also higher than that in the native ranges [44,45]. It was reported that total biomass and plant height of *C. odorata* obtained from invasive ranges were larger than those from the native ranges [41]. On the contrary, the aboveground biomass and plant height of *C. odorata* in the invasive ranges were smaller than those of the native ranges [39]. The aboveground biomass of *C. odorata* in the native ranges was 30% greater than that in the invasive ranges [46].

Plants produce large number of secondary metabolites in many chemical classes. The biosynthesis of some secondary metabolites is induced or synthesized de-novo under certain circumstances. Many of these secondary metabolites in the invasive plants have been reported to show multiple functions such as anti-herbivore, anti-fungal, anti-microbial, and allelopathic activity, and contribute to increasing the fitness of the plants in the invasive ranges [47,48,49,50,51,52,53,54,55,56]. Available information from a large number of publications suggests that *C. odorata* is allelopathic, and contains the compounds involved in the allelopathy. The plant species was also reported to show the defense response against the natural enemies such as herbivores and pathogens, and contain the compounds involved in the defense function. However, there has been no review paper focusing on the allelopathy and defense response of *C. odorata*, and compounds involved in these functions. This review provides an overview of the defense response and allelopathy of the species and compounds involved in the defense and allelopathy. Then, their possible involvement in the invasiveness of the species is discussed.

## 2. Interaction of *C. odorata* with the Natural Enemy

The interaction between the invasive plants and their natural enemies such as herbivores and pathogens, is one of the important factors for the naturalization of the invasive plants [29,30,31,57,58]. The population of *C. odorata* is controlled by many insects and pathogens in its native ranges [59,60]. More than 200 species of the herbivores were counted in the native ranges of *C. odorata*, and 25% of them are specific species in the native ranges [61]. There may be fewer specific herbivores in the invasive ranges. In fact, very few specific insect species for *C. odorata* in the invasive range (South Africa) were counted [62]. According to the evolution of increased competitive ability hypothesis, the success of the invasive species is due to fewer specialized predators in the invasive ranges. The invasive plants can allocate the resources from the high-cost defense strategy to the low-cost defense strategy and plant growth, leading to the successful naturalization [63,64].

### 2.1. Interaction of C. odorata with Insects

Powder of the roots, stems, and leaves of *C. odorata* increased the mortality of leaf beetle, *Callosobruchus maculatus* Fabricius [65,66]. Essential oil obtained from *C. odorata* leaves also increased the mortality of adult weevil, *Sitophilus zeamais* Motschulsky [67,68]. Aqueous leaf extract of *C. odorata* induced the increasing larval mortality of black fly, *Simulium* spp. [69], and an adult stage of cockroach, *Periplaneta americana* Linnaeus [70]. Aqueous ethanol leaf extracts of *C. odorata* were applied to *Aabelmoschus esculentus* (L.) Moench once a week for 4–7 weeks after its planning. The treatments resulted in the reduction of the population of whitefly, *Bemissa tabaci* Gennadius, and leafhopper, *Amrasca biguttula* Ishida on *Aabelmoschus esculentus* [71]. The methanol extracts of *C. odorata* leaves also showed ovicidal, antifeedant, and larvicidal activity on a leaf-eating insect, *Spodoptera litura* Fabricius [72]. These observations suggest that *C. odorata* possess anti-insect activity and contain certain compounds involved in the activity.

**Figure 2:**
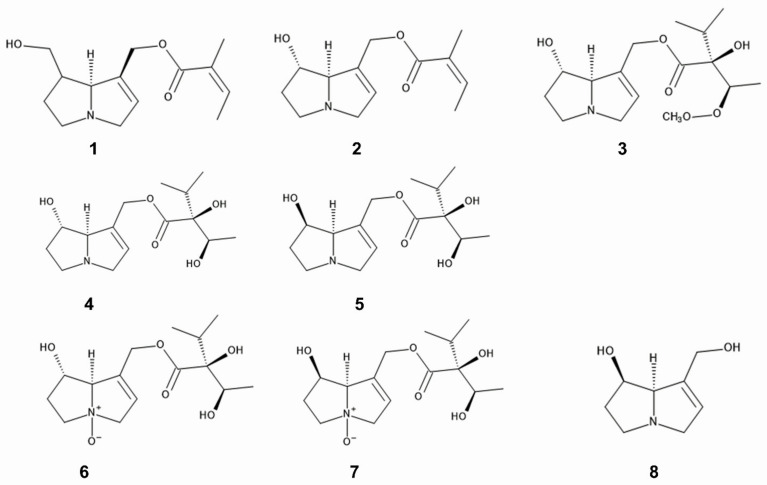
Pyrrolizidine alkaloids; 1: 7-angeloylretronecine, 2: 9-angeloylretronecine, 3: 3′-acetylrinderine, 4; rinderine, 5: intermedine, 6: rinderine *N*-oxide, 7: intermedine *N*-oxide, 8: 1,2-dehydropyrrolizine alkaloid.

Pyrrolizidine alkaloids such as 7- and 9-angeloylretronecine, intermedine, rinderine, and 3′-acetylrinderine were isolated from roots and mature flower heads of *C. odorata* [73], and rinderine *N*-oxide and intermedine *N*-oxide were identified in its roots [74] (Figure 2). Pyrrolizidine alkaloids are amino alcohols, esterified with mono- or dicarboxylic acids [75], and act as chemical defense agents against herbivores such as insects and mammals [76,77,78,79]. The compounds are highly toxic including hepatotoxicity, and disturb several metabolisms in the cell functions [80,81,82,83]. Therefore, these pyrrolizidine alkaloids in *C. odorata* may be involved in the anti-insect activity caused by the extracts and power of the species as describe above, and contribute to the protection of the species from herbivore attacks.

Some specialist insects obtain pyrrolizidine alkaloids from plants and store them. Those stored pyrrolizidine alkaloids are used for their protection from their predators as poison, and for the precursors to synthesize their mail pheromones [75,84,85]. However, *C. odorata* may scarcely meet these specialist insects in its invasive ranges since the host plants of the sspecialist insects are narrow and there may be no such co-evolutional history between *C. odorata* and the insects in the invasive ranges (Figure 2, Table 1).

### 2.2. Interaction of C. odorata with Nematodes

Plant parasitic nematodes such as *Meloidogyne* spp. (root-knot nematode), *Helicotylenchus* spp., and *Pratylenchus* spp. feed on the roots of plants, and their feeding process causes serious injuries and reduces the ability of the plants to absorb nutrients and water, leading to losing plant vigor and defense capability against other pathogen attacks [86,87,88]. The population density of nematodes such as *Meloidogyne* spp., *Helicotylenchus* spp. and *Pratylenchus* spp., was suppressed by the *C. odorata* infestation into the invasive ranges with various soil conditions [89]. It was also reported that *C. dorata* reduced by 77–81% of plant parasitic nematode population; *Meloidogyne* spp., *Helicotylenchus spp.,* and *Pratylenchus* spp. in the soils after two years invention [90]. *C. odorata* also suppressed the increasing population of *Meloidogyne incoginita* Kofoid & White in the pot experiments [91]. These observations suggest that some compounds may be released from *C. odorata* and accumulated in the soils, and these compounds may suppress the population of plant parasitic nematodes in the soils.

The incorporation of plant powder of *C. odorata* into the field soil prevented the increasing population of *Meloidogyne incoginita* [92]. Aqueous root extracts and root mulch of *C. odorata* showed the suppression of the parasitism of *Meloidogyne incoginita* into the roots of *Lactuca sativa* L. [93]. Therefore, certain compounds in the plant powder, roots, and extracts of *C. odorata* may work for the suppression.

1,2-Dehydropyrrolizine alkaloid was identified in the root extracts of *C. odorata* and the compound showed anti-nematode activity [93] (Figure 2). 1,2-Dehydropyrrolizine alkaloid was reported to be synthesized and stored in vacuole in the roots of *C. odorata* [94]. As described in the Section 2.1, pyrrolizidine alkaloids are highly toxic and act as chemical defense agents against natural enemies [76,77,78]. Those observations suggest that the extracts and powder *of C. odorata,* and the soil under *C. odorata may* suppress the population of the nematodes, and prevented the hatch and parasitism of the nematodes. Certain compounds including 1,2-dehydropyrrolizine alkaloid may cause the suppression and acts as anti-nematode agents of *C. odorata* (Table 2).

### 2.3. Interaction of C. odorata with Microbial

The invasion of *C. odorata* into the forest and savanna in West Africa increased the soil microbial activity, and the amount of available N and P in the soil. *C. odorata* altered the soil microbial community in the invasion ranges. The altered microbial community suppressed the growth of the native plant species such as *Eupatorium japonicum* Thunb. and *Eupatorium heterophyllum* DC., and stimulated the growth of *C. odorata* [95]. The population of an arbuscular mycorrhizal fungus *Paraglomus* spp. was also increased in the soil under *C. odorata* [96]. Arbuscular mycorrhizal fungi increase the ability of their host plants to absorb water and nutrients, and enhance the defense function against several stress conditions and pathogen attacks [97,98,99]. The observations suggest that certain compounds from *C. odorata* may alter the soil microbial community in the invasion ranges and the alteration may contribute the invasion of *C. odorata.* However, an arbuscular mycorrhizal fungus *Paraglomus* spp. colonizes with a wide range of plant species [100,101]. The colonization may occur with other plant species and promote their growth in the invasive ranges of *C. odorata*. In addition, the abundance of the arbuscular mycorrhizal colonization of *C. odorata* in its invasive range (South Africa) was reported to be 50% of its native ranges (Puerto Rico) [102].

The rhizosphere soil of *C. odorata* increased the population of the soil borne fungal pathogen, *Fusarium ssp.,* and inhibited the growth of *Amaranthus spinosus* L. and *Bambusa bambos* (L.) Voss. Sterilization of the soil eliminated these effects. The root leachate of *C. odorata* increased the spore density of *Fusarium* spp. in *C. odorata-*free soil. The increases were illuminated by adding activated carbon into the soil [103]. It was also reported that the root exudates of *Sorghum bicolor* (L.) Moench. and *Vigna unguiculata* (L.) Walp. increased the population of *Fusarium* spp. [104], and phenolics in the root exudates of *Glycine max* (L.) Merr. increased the population of *Fusarium* spp. [105]. The observations suggest that certain compounds in the root exudate of these plant species including *C. odorata* may stimulate the increasing population of *Fusarium* spp. However, it is not clear if the increased *Fusarium* spp. population affects the growth of *C. odorata.*

On the contrary, it was reported that the extracts of *C. odorata* suppressed the growth of some soil borne fungal pathogens including *Fusarium* spp. Aqueous methanol leaf extracts of *C. odorata* significantly suppressed the colony growth of the pathogens, *Lasiodiplodia theobromae* (Pat.) Griffon & Maubl. and *Lasiodiplodia pseudothobromae* A.J.L. Phillips, A. Alves & Crous [106]. Both *Lasiodiplodia* spp. are members of the Botryosphaeriaceae family and cause leaf necrosis, canker, and dieback in many plant species [107,108]. The methanol leaf extracts of *C. odorata* suppressed the growth of *Bacillus subtilis* Cohn, and *Bacillus cereus* Frankland & Frankland [109]. The ethanol plant extracts of *C. odorata* suppressed the growth of soil borne pathogen fungi, *Phytophthora colocasiae* Racib., and *Fusarium oxysporum* Schlecht. emend. Snyder & Hansen [110]. Acetone extracts of *C. odorata* also suppressed the colony growth of the pathogen fungi, *Pythium ultimum* Trow, *Rhizoctonia solani* J.G. Kühn, *Fusarium oxysporium* Schlecht. emend. Snyder & Hansen, and *Phytophthora nicotianae* Breda de Haan [111] and *Pyricularia oryzae* Cavara [112]. Essential oil of *C. odorata* suppressed the growth of *Rhizoctonia solani* J.G. Kühn, *Fusarium graminearum* Schwabe, *Exserohilum turcicum* (Pass.) K.J. Leonard & Suggs, *Botrytis cinereal* Pers., and *Sclerotinia sclerotiorum* (Lib.) de Bary [113]. These observations suggest that the extracts of *C. odorata* possess the anti-fungal activity, and may contain certain compounds involved in the activity.

Some compounds in the extracts and/or the rhizosphere soil of *C. odorata* may be involved in the alteration of the microbial community as the observations in those publications (Table 3). The identification of these compounds is also necessary. In addition, the observations described in this section are controversial that whether *C. odorata* increases the population of fungal pathogen such as *Fusarium* spp. or suppressed the population. More sophisticate investigations are necessary in the future to explain the interaction of *C. odorata* with the microbial population.

## 3. Allelopathy of *C. odorata*

The interaction between the invasive plants and the indigenous plant species in the invasive ranges is also one of the important factors for the naturalization of the invasive plants [30,31,48,50,51]. According to the novel weapons hypothesis, the competitive ability of the invasive plants against the indigenous plants are high because allelochemicals (weapons) released from the invasive plants inhibit the germination and growth of the indigenous plant species. The inhibitory effect of the allelochemicals was greater on the indigenous plant species in the invasive ranges than that on the neighboring plant species in the native ranges of the invasive plant species. These allelochemicals were new to the indigenous plant species in the invasive ranges. However, the co-evolutional history of the neighboring plant species with the invasive plant species allows for those neighboring plant species to gain the tolerance to these allelochemicals. Therefore, these allelochemicals are more effective on the indigenous plant species in the invasive ranges than the neighboring plant species in the native ranges of the invasive plants, and contribute to the invasions [48,49,50]

The seeds of *C. odorata,* which were obtained from the population in the native (Mexico) and invasive (China) ranges, were germinated and grown in a field in China under natural condition for 10 months. The biomass of *C. odorata* seeds obtained from the invasive range was greater than that from the native range. Both the *C. odorata* from Mexico and China were grown together with other plant species from Mexican origin and those from Chinese origin. The biomass of the *C. odorata* seed variety grown with Chinese original plant species was greater than that grown with Mexican original plant species, while these Mexican original plant species grew well with both *C. odorata* than the Chinese original plant species [114,115,116]. These observations suggest that the Mexican original plant species may have stronger resistance to *C. odorata* than the Chinese original plant species. The co-evolutional history of the neighboring plant species in the native ranges of *C. odorata* are longer than the plant species in the invasive ranges, and those neighboring plant species in the native ranges may have more competitive ability to *C. odorata* than the plant species in the invasive ranges. These observations may be consistent with the novel weapons hypothesis described above.

Allelopathy is the chemical interaction between donner plants and their neighboring plants through certain secondary substances defied as allelochemicals. Allelochemicals are synthesized in the donner plants and released into the vicinity of the donner plants either by root exudation, volatilization, rainfall leachates or decomposition of plant residues and litter. Since allelochemicals are thought to be stored in certain plant tissues until their releasing into the vicinity of donner plants [117,118,119,120], many researchers determined the allelopathic activity of *C. odorata* in its plant residues and extracts from different plant parts of *C. odorata* (Table 3).

### 3.1. Allelopathic Activity of the Residue of C. odorata

The incorporation of *C. odorata* leaves into the crop field soil resulted in the suppression of the growth of *Capsicum annuum* L. and *Solanum melongena* L. [121]. The leaf residues of *C. odorata* incorporated into soil under greenhouse conditions inhibited the growth of *Eleusine indica* (L.) Gaertn., *Cyperus iria* L. and *Ageratum conyzoides* L. [122], and *Lycopersicon esculentum* Mill. [123]. The leaf powder of *C. odorata* also suppressed the growth of *Crassocephalum crepidioides* (Benth.) S. Moore [124]. These observations suggest that *C. odorata* leaves contain some allelochemicals, and those allelochemical were released into the soil during their decomposition process.

### 3.2. Allelopathic Activity of the Extracts of C. odorata

Aqueous extracts of leaves and roots of *C. odorata* suppressed the germination and growth of five plant species from the native ranges of *C. odorata* such as *Bidens pilosa* L. (tropical America origin), *Ageratum conyzoides* L. (tropical America), *Amaranthus spinosus* L. (tropical America), *Conyza sumatrensis* (Retz.) E. Walker (South America), and *Chenopodium ambrosioides* L. (Central and South America), and other five plant species from the invasive ranges such as *Rottboellia exaltata* (Lour.) Clayton (tropical Africa and Asia), *Digitaria sanguinalis* (L.) Scop. (South Europa, North Africa and Asia), *Hemisteptia lyrata* Bunge. (Eurasia and north Africa), *Youngia japonica* (L.) DC. (Eastern Asia), and *Dicliptera chinensis* (L.) Juss. (Eastern Asia). Growth inhibitory effect on these ten test plant species was higher with the leaf extracts than the root extracts of *C. odorata*, and on plant species from the invasive ranges than that from native ranges of *C. odorata* [125]. It was also reported that the growth inhibitory activity of the leaf extracts of *C. odorata* collected from invasive ranges (China) was higher than that collected from native ranges (Mexico) against the growth of the indigenous plant species in China; *Eupatorium japonicum* Thunb., *Eupatorium stoechadosmum* Hance and *Eupatorium lindleyanum* DC [114]. Those observations suggest that the inhibitory activity of *C. odorata* extracts from the invasive ranges was greater than that from the native ranges, and the plant species from its invasive ranges were more susceptible to the extracts than those from the native ranges of *C. odorata*.

The seeds of *Lathyrus sativus* L. were dipped in the aqueous leaf extract of *C. odorata* for 8 h, washed with distill water, and dried. Then, the seeds were sown and the germination and growth of *Lathyrus sativus* were determined after 10 days and 30 days, respectively. The treatments resulted in the suppression of the germination and the growth of *Lathyrus sativus* [126]. The observation suggests that some allelochemicals may be absorbed into the seeds and suppress the seed germination and growth processes.

Aqueous leaf extracts of *C. odorata* showed the suppression of the germination of weed plant species, *Cynodon dactylon* L., *Crassocephalum crepidioides* (Benth.) S.Moore, and *Ageratum conyzoides* L. [124], and the growth of *Eleusine indica* (L.) Gaertn., *Cyperus iria* L., and *Ageratum conyzoides* L. [122]. Aqueous extracts of whole plants of *C. odorata* inhibited the germination and growth of *Echinochloa crus-galli* (L.) P.Beauv., and *Amaranthus viridis* L. [127].

Aqueous leaf extracts of *C. odorata* also inhibited the germination and growth of crop plant species, *Sorghum bicolor* (L.) Moench, *Zea mays* L., *Phaseolus vulgaris* L., *Vigna radiata* (L.) R.Wilczek, and *Centrosema pubescens* Benth. [128,129,130], *Brassica chinensis* L. [131], *Glycine max* L., and *Gossypium hirsutum* L. [132], and *Lycopersicum esculentum* Mill. [123]. Aqueous extracts of the aerial parts of *C. odorata* inhibited the germination and growth of *Sesame indicum* L., *Brassica nigra* (L.), *Brassica juncea* (L.) Czern., and *Raphenus raphanistrm* L. [133]. The leaves of *C. odorata* were extracted with methanol, and the extract was sprayed to *Amaranthus spinosus* L. and *Amaranthus spinosus* L. The treatments resulted in the reduction of the growth parameters of both plant species such as their plant high, leaf areas, root length, and plant masses [134].

These observations suggest that the aqueous and methanol extracts of all parts of *C. odorata* possess allelopathic activity on the germination and growth of both weed and crop plant species, and may contain water and/or methanol extractable allelochemicals. Effectiveness of these allelochemicals was greater on the plant species from the invasive ranges than the native ranges of *C. odorata*.

### 3.3. Mechanism of the Inhibition

The inhibitory mechanism of the extracts of *C. odorata* on the germination and growth of several plant species was also investigated. Aqueous leaf extracts of *C. odorata* suppressed amylase activity in the seeds of *Cicer arietinum* L., and *Cajanus cajan* (L.) Millsp. [135]. Aqueous extracts of leaves, stems, and roots of *C. odorata* were sprayed onto 21-day-old plants of *Ageratum conyzoides* L. every two days for 28 days. The treatments resulted in the reduction in the contents of chlorophyll, carotenoids, and stomata number in the leaves of *A. conyzoides* [136]. The leaves of *C. odorata* were soaked in water for 48 h, and the soaking water also reduced the contents of chlorophyll and protein, and cell division of *Allium cepa* L. [137]. The hexane fraction obtained from the aqueous methanol leaf extracts of *C. odorata* suppressed chlorophylls and carotenoid contents in the leaves of *Echinochloa crus-galli* (L.) P.Beauv. [127]. These observations suggest that the extracts disturb amylase activity in the seeds, cell division, and reduce the pigments of photosynthesis, which may cause the suppression of the photosynthesis and growth. The induction of amylase is essential for seed germination because this enzyme triggers starch degradation of the reserve starch in seeds and enable the seeds to germinate and grow [138,139]. Total concentrations of terpenoids, flavonoids, tannins, steroids, and alkaloids were determined in the aqueous extracts of *C. odorata* (Hamidi et al., 2014). Total terpenoids, flavonoids, phenols, and steroids were also determined in the ethanol extracts of *C. odorata* [134]. However specific compounds did not identify in these extracts. The observations described in this section suggest that the extracts of *C. odorata* are allelopathic and contain allelochemicals. Allelochemicals in the extracts of *C. odorata* should be identified in the furfure. Table 4 shows the allelopathic activity of *C. odorata* described in the Section 3.

## 4. Diversity of the Secondary Metabolites in *C. odorata*

Some of the plant secondary metabolites function as defense molecules against herbivores, pathogens, and competing neighboring plants. These compounds are important for the plant’s survival and fitness, and represent the adaptive characters of the plant species that have been subjected to natural selection during the evolution. The pattern of the plant secondary metabolites is complex, and changes in an organ- and tissue-specific way, and during the developmental stages of the plants. The biosynthesis of some secondary metabolites is induced or synthesized de-novo upon herbivore-attack, pathogen–infection and competition with neighboring plants [117,140,141,142,143,144].

The significant difference in the metabolomic profiles between *C. odorata* obtain from the native ranges (USA) and that from the invasive ranges (South Africa) were observed by the ultra-performance liquid chromatography-mass spectrometry. The major differences were high concentrations of flavonoids and flavone glycosides in the *C. odorata* from the invasive ranges comparing to those from the native ranges [145]. Although the function of these metabolites and their significance to the invasive behaviors of *C. odorata* are not apparent, the observation suggests that the invasive plant species may employ the metabolic flexibility and/or rapid adaptive evolution to succeed as the invasive plant species.

**Figure 3:**
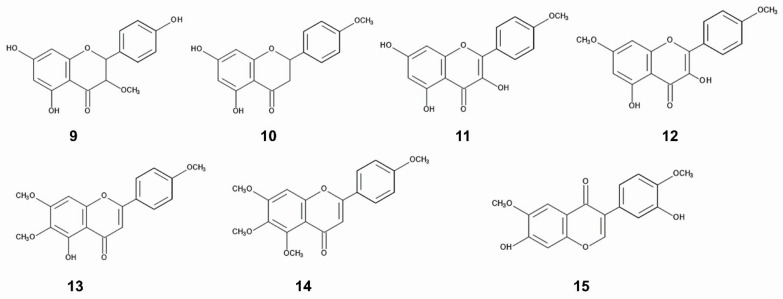
Flavonoids; 9: dihydrokaempferol-3-methoxy ether, 10: isosakuranetin, 11: kaempferide-4’-methoxy ether, 12: 3,5-dihy-droxy-7,4’-dimethoxyflavone, 13: acutellerin-4’,6,7-trimethy ether, 14: 4’,5,6,7-tetramethoxyflavone, 15: odoratin. The concentration of these compounds was grater in *C. odorata* collected from the invasive ranges than that from the native ranges.

As already described in the Section 3.2, the inhibitory activity of the extracts of *C. odorata* collected from invasive ranges was higher than that collected from the native ranges [114,125]. Total phenolic concentration in the leaves and stems of *C. odorata* obtained from the invasive ranges (China) was greater than that from the native ranges (Mexico) [39]. Concentrations of flavonoids; dihydrokaempferol-3-methoxy ether, isosakuranetin, kaempferide-4’-methoxy ether, 3,5-dihy-droxy-7,4’-dimethoxyflavone, acutellerin-4’,6,7-trimethy ether and 4’,5,6,7-tetramethoxyflavone were also greater in *C. odorata* in the invasive ranges (China, Laos, Thailand, Vietnam, Philippines, Sri Lanca, Malaysia) than those in the native ranges (USA, Mexico, Puerto Rico, Trinidad, and Tobago) [41]. The concentration of a flavonoid, odoratin in *C. odorata* obtained from an invasive range was 2.4-fold greater than that from the native ranges. However, the specific compounds of the increasing phenolics were not identified. The function of these flavonoids on the allelopathy of *C. odorata* is also not clear. In addition, the chemical structure of odratin in the publication is not correct [46] (Figure 3).

Pharmacological investigations showed that *C. odorata* contains secondary metabolites in many chemical classes, such as flavonoids, phenolic acids, saponins, terpenoids and tannins. Some of those compounds were related to the pharmacological activity such as analgesic, antipyretic, anti-inflammatory, anti-diabetic, anticancer, and antioxidant activity [146,147,148,149,150,151,152,153,154]. Although most of those identified compounds have not yet been related to the invasiveness of the plant species, some of them may be involved in the allelopathy and defense functions against herbivores, nematodes, and fungal pathogens.

**Figure 4:**
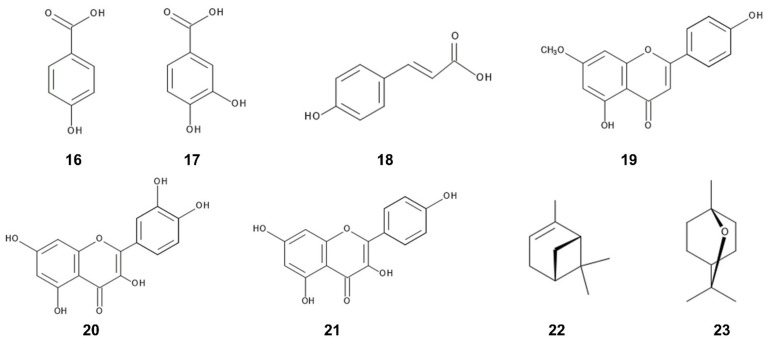
Potential allelochemicals; 16: *p*-hydroxybenzoic acid, 17: protocatechuic acid, 18: *p*-coumaric acid, 19: sakuranetin, 20: quercetin, 21: kaempferol, 22: α-pinene, 23: 1,8-cineole.

Benzoic acid and cinnamic acid derivatives such as *p*-hydroxybenzoic acid, protocatechuic acid, and *p*-coumaric acid were identified as the major compounds in the leaves of *C. odorata* [146]. These compounds are synthesized by shikimic acid pathway [155,156]. Benzoic acid and cinnamic acid derivatives have been found in a wide range of plants, decomposition products of plants, and plant rhizosphere soil. The involvement of those compounds in allelopathy and their mechanisms of the action have been investigated in other plant species [157,158,159]. Benzoic acid and cinnamic acid derivatives reduced the transmembrane electrochemical potential of the plasma membrane of the cells. The depolarization of the membranes caused a nonspecific efflux of both cations and anions, and affected the membrane permeability and the uptake of ions and nutrients. These compounds caused structural alteration in the membranes including a variety of membrane proteins. The changes in ion flux through the membranes affected plant water status including the stomatal functions. These compounds also suppressed several enzyme activities involved in several physiological processes such as respiration, phytohormone synthesis, protein synthesis, and synthesis of some other secondary metabolites [157,158,159]. In addition, their derivative, chlorogenic acid was also isolated from the *C. odorata* leaves [160]. Chlorogenic acid was reported to inhibit blue-green alga *Microcystis aerugonisa* Kützing [161] (Figure 4).

Several flavonoids were identified in the leaf and flower extracts of *C. odorata* [147,149,150,152]. Flavonoids are polyphenolic secondary metabolites having a 15-carbon skeleton, consisting of two benzene rings and a heterocycling ring, and synthesized from chalcone. Many of the flavonoids showed anti-herbivore, anti-fungal, and anti-bacteria activity [162,163,164]. Sakuranetin was isolated from the leaf extracts of *C. odorata* [165]. The compound is known to act as phytoalexin against pathogen infection [166,167]. Quercetin and kaempferol were also identified in the leaf extracts *C. odorata* [145,168] and both compounds were reported to work as allelopathic agents in the other plant species. Quercetin inhibited the growth of several pant species [169,170], and the plant mitochondrial function [171,172]. Kaempferol showed the growth inhibitory activity on blue-green alga *Microcystis aerugonisa* Kützing [173]. Kaempferol reduced the efficiency of the photosystem II in the chloroplasts [171] (Figure 4).

Several terpenoids were identified in the essential oil and leaf extracts of *C. odorata* [68,148]. Terpenoids in the most plants are synthesized through the mevalonate pathway from acetyl-CoA, and many terpenoids were reported to be involved in the defense function of plants such as anti-fungal, anti-bacterial, and anti-feeding activities, and in the interaction with insects such as for the pollination, and the attracting predators of their natural enemy [174,175,176,177,178,179]. The essential oil mixture, of which major constituents were monoterpenes and sesquiterpens, showed allelopathic activity [180,181,182]. Among terpenoids found in *C. odorata*, monoterpenes, α-pinene and 1,8-cineole were identified in its leaf extracts [68]. α-Pinene and 1,8-cineole showed allelopathic activity and a mixture of both compounds exhibited synergistic effect for the allelopathic activity on the growth of *Solanum elaeagnifolium* Cav. [183]. 1,8-Cineole strongly inhibits the roots and coleoptile growth of *Echinochloa crus-galli* (L.) P.Beauv. and *Senna obtusifolia* (L.) H.S.Irwin et Barneby, and reduced all stages of mitosis of the root tips of *Allium cepa* L. [184] (Figure 4). Several pyrrolizine alkaloids were also identified in the leaf extracts of *C. odorata* and showed anti-herbivore and anti-nematode activity as already described in the Section 2.1.

As describe above, some of cinnamic acid and benzoic acid derivatives, flavonoids, and terpenoids found in *C. odorata* may affect the physiological processes of the neighboring plant species, causing growth inhibition and reducing their fitness as allelochemicals. Pyrrolizine alkaloids found in *C. odorata* may also have defense functions against herbivores, and pathogenic fungi, microbes, and nematodes. Therefore, these compounds may contribute to the invasiveness and naturalization in the introduced rages of *C. odorata.*

## 5. Conclusions

*C. odorata* is highly invasive and has naturalized in many countries. The paper described the interaction between the species and their natural enemies, and the species and the indigenous plant species in the invasive ranges for the naturalization of the invasive plants. The extracts of all plant parts, residues, and rhizosphere soil of *C. odorata* showed the inhibitory activity against various insects, parasitic nematodes, and fungal pathogens, and several pyrrolizidine alkaloids were identified in the roots and flower heads of *C. odorata*. Pyrrolizidine alkaloids are toxic against insects and other organisms. Therefore, pyrrolizidine alkaloids of *C. odorata* may act as chemical protection agents against natural enemies including herbivores.

The rhizosphere soil, extracts, and residues of all plant parts showed the suppression of the germination of growth of many plant species including indigenous plant species in the invasive ranges. The indigenous plant species from the invasive ranges of *C. odorata* were more susceptible to the extracts than plant species from the native ranges of *C. odorata*. Those observations suggest that *C. odorata* possess allelopathic activity, and may contain certain allelochemicals. These allelochemicals may be more effective on the plant species from the invasive ranges of *C. odorata* than on the plant species from its native ranges.

Pharmacological investigations showed that *C. odorata* contains secondary metabolites in many chemical classes, such as phenolic acids, flavonoids, saponins, tannins, and terpenoids. Although many of these compounds have not been related to the allelopathy of *C. odorata*, benzoic acid and cinnamic acid derivatives such as *p*-hydroxybenzoic acid, protocatechuic acid, and *p*-coumaric acid; flavonoids such as sakuranetin, quercetin and kaempferol; and monoterpenes such as α-pinene and 1,8-cineole identified in the leaves of *C. odorata* were reported to be involved in the allelopathy of other plant species. Therefore, these compounds may also work as allelopathic agents for *C. odorata* and inhibit the germination and growth of the neighboring plant species.

These characteristics of *C. odorata* for the defense function against their natural enemies such as insects, parasitic nematodes, and fungal pathogens, and allelopathic potential may contribute to the invasiveness and naturalization of *C. odorata* in the new habitats as invasive plant species. However, allelochemicals are able to work only when they are released into the neighboring environment of the donner plants either by root exudation, volatilization, rainfall leachates, or decomposition of plant litter and residues. Therefore, it is necessary to determine the concentration of these allelochemicals in the neighboring environment of *C. odorata* such as the rhizosphere soil.

## Figures and Tables

**Table 1:** Interaction *of C. odorata* with insects.

Source	Insect	Action	Reference
Root powder	*Callosobruchus maculatus*	Mortality (100%) at 8% (weight of powder/weight of crop grain)	[65]
Leaf powder	*Callosobruchus maculatus*	Mortality (51%) at 1.7% (weight of powder/weight of crop grain)	[66]
Essential oil from leaves	*Sitophilus zeamais*	Mortality (100%) at 0.5% (volume of essential oil/weight of crop grain)	[67]
Leaf extract	*Sitophilus zeamais*	Mortality (50%) at 40 μg/mL methanol extract	[68]
	*Simulium* spp.	Mortality (100%) at 1 mg dry mass of aqueous extract/mL	[69]
	* Periplaneta americana *	Mortality (36%) at 1 mL dose of 20% (*w*/*v*) aqueous extract	[70]
	*Bemissa tabaci*, *Amrasca biguttula*	Population 25–50% decrease of control at 2.5% (*w*/*v*) ethanol extract	[71]
	*Spodoptera litura*	Mortality 68% at 5% (*w*/*v*) methanol extract	[72]

**Table 2:** Interaction *of C. odorata* with nematodes.

Source	Nematode	Action	Reference
Plant infestation soil	*Meloidogyne* spp., *Helicotylenchus* spp., *Pratylenchus spp.,*	Population decrease by 67–79% of control after 30 months	[89]
	*Meloidogyne* spp., *Helicotylenchus* spp., *Pratylenchus spp.,*	Population decrease by 77–81% after 24 months	[90]
	*Meloidogyne incoginita*	Population decrease by 92% after 2 months	[91]
Plant residues	*Meloidogyne incoginita*	Population decrease significantly at 1% (weight of powder/weight of soil)	[92]
Root extract	*Meloidogyne incoginita*	Parasitism suppression by 0.4–65% of control at 0.1–5% (*w*/*v*) aqueous extract	[93]
Root mulch	*Meloidogyne incoginita*	Parasitism suppression by 0–22% at 0.1–5% (weight of plant/weight of soil)	[93]

**Table 3:** Interaction of *C. odorata* with microbial population.

Source	Species	Population	Reference
Plant infestation soil	*Paraglomus* spp.	Increase biomass significantly after 10 months incubation	[96]
Rhizosphere soil	*Fusarium* ssp.	Increase spore number 25 times after 5 days incubation	[103]
Leaf extract	*Lasiodiplodia theobromae, Lasiodiplodia pseudothobromae*	Decrease biomass by 6–17% of control at 3% (*w*/*v*; extract residue/medium)	[106]
	*Bacillus subtilis, Bacillus cereus*	Decrease population significantly at 10% (*w*/*v*; plant weight/solvent)	[109]
	*Phytophthora colocasiae, Fusarium oxysporum*	Decrease of the colony expansion by 20–33% of control at 100 mg leaves/mL methanol	[110]
	*Pythium ultimum, Rhizoctonia solani, Fusarium oxysporium, Phytophthora nicotianae*	Inhibitory activity at concentration of 0.09 mg residue of extract/mL of medium	[111]
	*Pyricularia oryzae*	Decrease by 12–43% of control at 10% (*w*/*v*; extract residue/solvent)	[112]

**Table 4:** Allelopathic activities of *C. odorata*.

Source	Inhibition				Target Plant Species	Reference
	Germination	Growth	Chlorophyll	Amylase		
Plan residue		✔			*Capsicum annuum, Solanum melongena*	[121]
	✔	✔			*Eleusine indica, Cyperus iria, Ageratum conyzoides*	[122]
		✔			*Lycopersicon esculentum*	[123]
Leaf powder		✔			*Crassocephalum crepidioides*	[124]
Extract						
Leaf		✔			*Eupatorium japonicum, Eupatorium stoechadosmum, Eupatorium lindleyanum*	[114]
	✔	✔			*Lathyrus sativus*	[126]
	✔	✔			*Cynodon dactylon, Crassocephalum crepidioides, Ageratum conyzoides*	[124]
	✔	✔			*Eleusine indica, Cyperus iria, Ageratum conyzoides*	[122]
	✔	✔			*Sorghum bicolor, Zea mays, Phaseolus vulgaris, Vigna radiata, Centrosema pubescens*	[128,129,130]
	✔	✔			*Brassica chinensis*	[131]
	✔	✔			*Glycine max, Gossypium hirsutum*	[132]
		✔			*Lycopersicum esculentum*	[123]
	✔	✔			*Amaranthus spinosus, Amaranthus spinosus*	[134]
				✔	*Cicer arietinum, Cajanus cajan*	[135]
			✔		*Ageratum conyzoides*	[136]
			✔		*Allium cepa*	[137]
Leaf, root	✔	✔			*Bidens pilosa, Ageratum conyzoides, Amaranthus spinosus, Conyza sumatrensis, Chenopodium ambrosioides, Rottboellia exaltata, Digitaria sanguinalis, Hemisteptia lyrata, Youngia japonica, Dicliptera chinensis*	[125]
Aerial part	✔	✔			*Sesame indicum, Brassica juncea, Raphenus raphanistrm*	[133]
Whole plant	✔	✔	✔		*Echinochloa crus-galli, Amaranthus viridis*	[127]
